# The hallux valgus angle of the margo medialis pedis as an alternative to the measurement of the metatarsophalangeal hallux valgus angle

**DOI:** 10.1186/1471-2474-15-133

**Published:** 2014-04-21

**Authors:** Christian Klein, Wieland Kinz, Alexander Zembsch, Elisabeth Groll-Knapp, Michael Kundi

**Affiliations:** 1 EMCO Clinic Bad Dürrnberg, Prof. Martin Hell Str. 7-9, 5422 Bad Dürrnberg, Austria; 2 Institute of Environmental Health, Medical University of Vienna, Center for Public Health, Kinderspitalgasse 15, 1090 Vienna, Austria; 3 Center of Orthopedic Surgery Vienna-Hietzing, Hietzinger Hauptstraße 34, 1130 Vienna, Austria

**Keywords:** Hallux valgus angle, Radiographic measurement, Margo medialis pedis

## Abstract

**Background:**

Currently, the metatarsophalangeal angle (hallux valgus angle) is measured based on radiographic images. However, using X-ray examinations for epidemiological or screening purposes would be unethical, especially in children. For this reason it is discussed to measure the hallux valgus angle of the margo medialis pedis (medial border of the foot) documented on foot outline drawings or foot scans. As a first step on the way to prove the validity of those approaches this study assesses the hallux valgus angle measured on the margo medialis pedis based on the same x-ray pictures as the metatarsophalangeal hallux valgus.

**Methods:**

Radiographic images of the foot were obtained from patients with symptomatic hallux valgus malformation. Twelve sets of contact copies of the 63 originals were made, and were marked and measured according to three different methods, each one performed by two observers and with two repeated measurements. Thus, data sets from 756 individual assessments were entered into the multifactorial statistical analysis.

Comparisons were made between the angle of the margo medialis pedis and the metatarsophalangeal angle, which was determined by two different methods. To determine the inter- and intraobserver reliability of the different methods, each assessment was conducted by two independent experts and repeated after a period of several weeks.

**Results:**

The correlations between the hallux valgus angles determined by the three different methods were all above r = 0.89 (p < 0.001) and thus highly significant. The values obtained by measuring the margo medialis pedis angle, however, were on average 4.8 degrees smaller than the metatarsophalangeal angles. No significant differences were found between the observers. No systematic deviations for any observer between repeated measurements were detected.

**Conclusions:**

Measurements of the radiographic hallux angle of the margo medialis pedis are reliable and show high correlation with the metatarsophalangeal angle. Because the hallux valgus angles based on margo medialis pedis measurements were slightly but statistically significantly smaller, these measurements should be considered conservative estimates of the metatarsophalangeal angle. Significant differences between hallux valgus angles based on radiographic and non-radiographic material are unlikely. However this question has to be treated in a second stage in detail.

## Background

Hallux valgus malformation is one of the most common forefoot disorders. In a meta-analysis Nix showed a pooled prevalence for Hallux valgus in adults aged 18–65 years of 23% and in elderly people aged over 65 years of 35%. Prevalence increased with age and was higher in females compared to males [1].

The aetiology of hallux valgus deformities is complex. Besides intrinsic factors extrinsic factors are also involved. Diagnosis and treatment algorithms depend, among other factors, on the hallux valgus angle [2]. The hallux valgus angle is also important for mass-screening and preventive health studies [3]. Clinically, the valgus position of the great toe is routinely determined by measuring the angle created by the intersection of the lines that longitudinally bisect the proximal phalanx and first metatarsal drawn on traditional radiographic film or using digital imaging techniques. A number of different methods of measuring the metatarsophalangeal angle are described in the literature [4-11]. However, in addition to method-related differences, considerable intra- and interobserver variability has been reported [12].

For screening examinations radiographic imaging is not feasible for ethical reasons. Alternative methods, including measuring the hallux valgus angle of the margo medialis pedis on foot outline drawings foot prints or foot scans, are used in these cases [3]. Until now, the question of whether measurements of the hallux valgus angle of the margo medialis pedis are a suitable alternative to x-ray examinations of the metatarsophalangeal angle has not been systematically investigated. Without providing further methodological details, Barnicot and Hardy [13] observed a moderate correlation of r = 0.56 between hallux valgus angle measurements based on foot outline drawings or on radiographs. No other investigation of this issue was found.

Several other approaches for the measurement of the hallux valgus angle for epidemiological reasons have been reported [14-18]. The most developed of these tools are the Manchester scale described by Garrow and a line drawing tool described by Roddy [14,18]. A significant limitation of both methods is that a precise measurement of the deviation in degrees compared to radiological measurements is not possible. Nix reported measurements of hallux valgus by using standardized digital photographs [17]. Compared to clinical scales a more incremental measurement of the deviation is possible. But determining referent points on the photographs may be difficult.

The current study was conducted to assess whether measurements of the hallux valgus angle of the margo medialis pedis is a viable alternative to measurements of the metatarsophalangeal angle when taken from the same x-ray picture. This strategy guarantees absolutely identical external conditions (weight-bearing, foot position, temperature) which might influence the hallux valgus angle. After clarifying this fundamental question, possible differences between radiographic and non-radiographic margo medialis pedis measurements should be assessed in further investigations. Two different measurement procedures for the assessment of the metatarsophalangeal angle were used in this study because different measurement methods are applied in clinical routine. Particular care was taken to apply a standardised procedure since it is well documented that different methods of determining the axis of the first metatarsal and the proximal phalanx can result in considerable deviations in the measured angle [9,10,12]. Furthermore, inter- and intraobserver reliability was assessed for all methods.

## Methods

Thirty nine patients with a symptomatic hallux valgus malformation treated in the medical practice of one of the authors (CK) or in the orthopaedic out-patient clinic of EMCO Private Hospital were included in this study. All images were taken as part of standard preoperative patient care. No image was taken for research purposes only. In 24 subjects the deformity was bilateral. A total of 63 feet were measured, 31 right and 32 left feet. Twenty-seven patients were female and 12 male. The mean age was 55.6 years (21 – 84 years). According to Austrian law (Ethic Committee Salzburg) it was not mandatory for studies of this type to apply for a vote of the ethics committee. However an informed consent for participation and the use of pictures was signed.

Radiographic images were obtained applying standard techniques at two institutes (EMCO Private Hospital, Radiology Institute Dr. Doringer). X-ray images were taken unilaterally in a weight-bearing, standing position, with a film-focus distance of 100 cm and a tube tilt angle of 15 degrees [11,19]. The central beam was centred on the second tarsometatarsal joint. Lighting strength was chosen to ensure that beside the bony structures also the soft tissues of the foot would be sufficiently represented for providing also a clear picture of the foot outline and the margo medialis pedis comparable to foot prints, foot scans or foot outline drawings.

**Figure 1:**
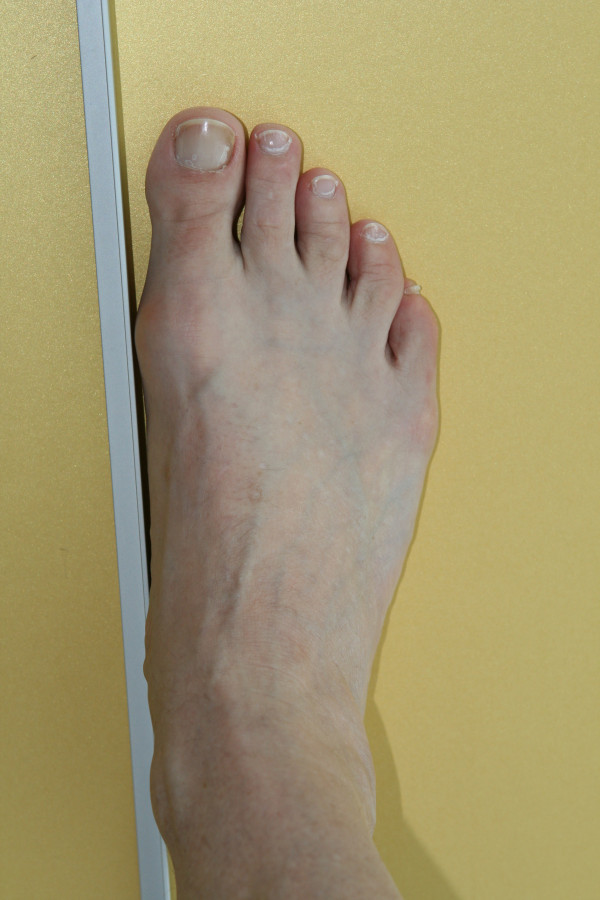
Metal strip placement for the x-ray imaging of the margo medialis pedis.

To determine the medial edge angle on the x-ray images, a metal strip was placed alongside the margo medialis pedis before the image was taken, running from the medial point of the heel (not pictured in the standard x-ray images) to the ball of the great toe (Figure 1).

Twelve sets of contact copies of the 63 originals were made, each with a unique identification number. Two experienced observers measured each radiograph by applying the method indicated by the code number. By this method each radiograph was independently measured applying three methods two times with an interval of at least 3 weeks by two observers (3 × 2 × 2 = 12 measurements). All auxiliary lines necessary to determine the hallux valgus angle were drawn by the individual observers, and the resulting angle was then obtained to within 0.5 degrees accuracy.

The following three methods of measurement were applied:

Method 1: Margo medialis pedis (MMP)

**Figure 2:**
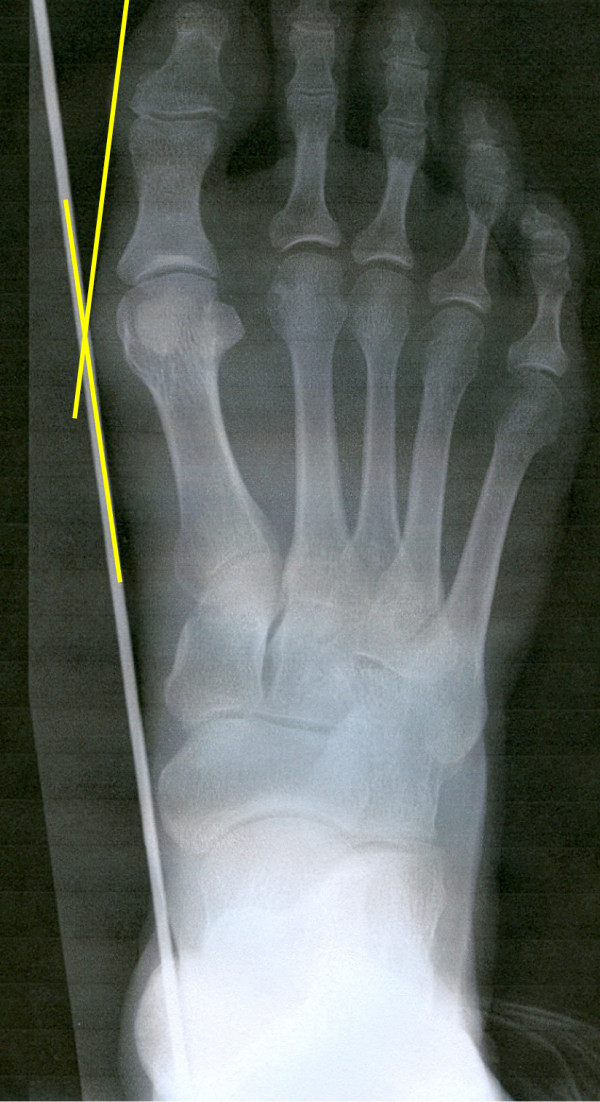
Margo medialis pedis (MMP) method to determine hallux valgus angle.

The hallux valgus angle is defined as the angle between the straight line created by the metal strip included on the x-ray images, connecting the medial point of the heel to the ball of the great toe, and a further straight line drawn on the image connecting the ball of the great toe to the most medial soft tissue shadow of the great toe (Figure 2). This procedure gives the possibility to assess the hallux valgus angle of the margo medialis pedis and the metatarsophalangeal angle from the same x-ray images with identical foot position and weight bearing condition.

Method 2: Centre base - centre head (CC) [6]

**Figure 3:**
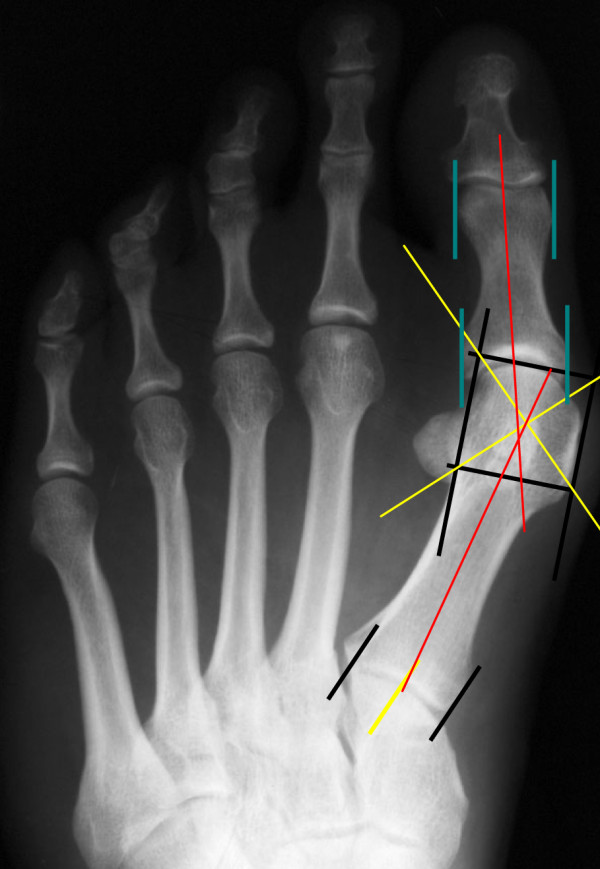
Centre Head – Centre base (CC) measuring method, pre- and postoperative site.

First the axis of the proximal phalanx was determined. The axis of the proximal phalanx was defined as the connecting line between the centre of the proximal joint area and the centre of the distal diaphysis. Two parallel lines were drawn on the medial and lateral borders of the head. The distal border is perpendicular to these lines on the distal joint area thus defining a square its diagonals defining the centre of the head. The second reference point is obtained by bisecting the base of the first metatarsal. Connecting these two points results in the axis of the first metatarsal (Figure 3). This construction for defining the centre was chosen such that the reference point disregards the cartilaginous joint surface area of the metatarsal head and could also be identified on post-operative x-ray images after removal of the medial exostosis. The postoperative x-rays were not included in this study.

Method 3: Shaft bisection (SB) [10]

**Figure 4:**
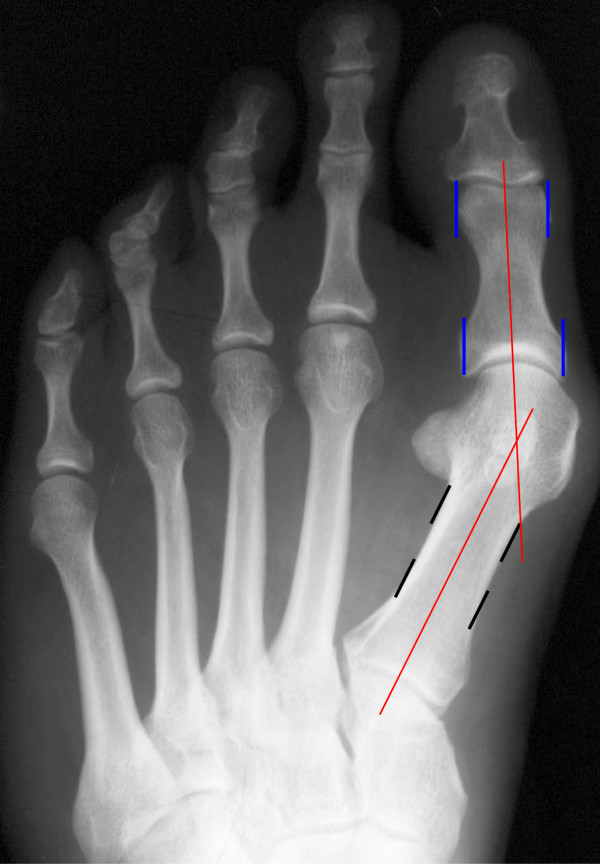
Shaft bisection (SB) measuring method.

Before the lines were drawn on the first metatarsal, the axis of the proximal phalanx was determined as in method 2. For determining the metatarsal axis, the first metatarsal shaft was bisected at two points in the diaphysis area [10]. The connection of these two points defined the metatarsal axis (Figure 4).

### Study design and statistical evaluation

As outlined above, the study applied a repeated measurement design with two observers, independent duplicate measurements, and three methods. Reliability of each method was assessed by intraclass correlation coefficients. Precision and accuracy (bias correction factor) of the estimates based on the margo medialis pedis method was determined by computation of concordance correlation coefficients. Also inter- and intraobserver agreement was determined by concordance correlation coefficients. For method comparisons Deming regression was performed with determination of the coefficient of variation using the duplicate measurements. 95% limits agreement (LA95%) were calculated to determine the range of differences between MMP and CC method as well as between MMP and SB method [20,21].

## Results

Average hallux valgus angles and standard deviations for all methods as well as coefficients of variation and reliability determined by intraclass correlation coefficients are shown in Table 1. The absolute range of the hallux angle was 5° to 67° within the CC method, 2° to 67° within the SB method and 0° to 63° within the MMP method.

**Table 1:** Average hallux valgus angle: results from the three measurement methods

**Method**	**Mean (°)**	**SD (°)**	**Coeff. of variation (%)**	**Reliability**
**MMP**	12.9	9.0	7.7	0.989
**CC**	17.7	9.3	8.6	0.973
**SB**	17.5	9.3	10.2	0.962

Hallux valgus angle as determined by the margo medialis pedis (MMP) method, the centre base – centre head (CC) method, and the shaft bisection (SB) method. Mean, standard deviation (SD), coefficient of variation, and reliability are shown.

**Figure 5:**
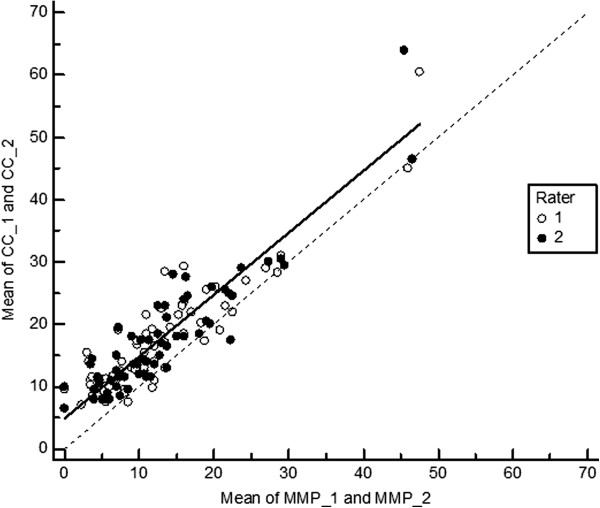
**Deming regression of hallux valgus angles measured by the centre base – centre head (CC) method on measurements based on the margo medialis pedis (MMP) method – measurements 1 and 2 – for both observers (solid line**). The dotted line indicates regression line for unbiased measurements.

**Figure 6:**
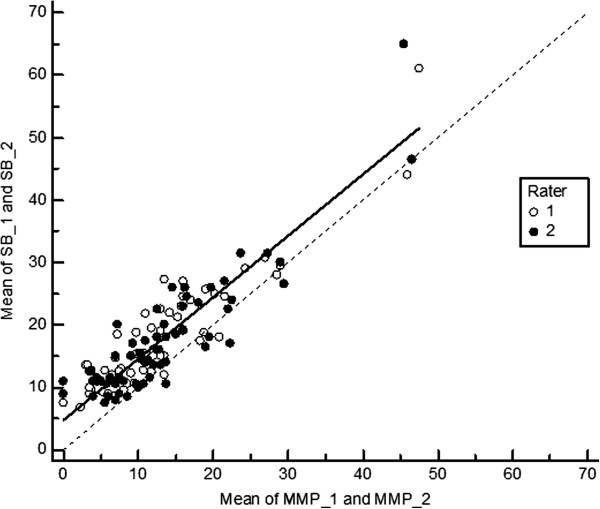
**Deming regression of hallux valgus angles measured by the shaft bisection (SB) method on measurements based on the margo medialis pedis (MMP) – measurements 1 and 2 – for both observers (solid line).** The dotted line indicates regression line for unbiased measurements.

**Figure 7:**
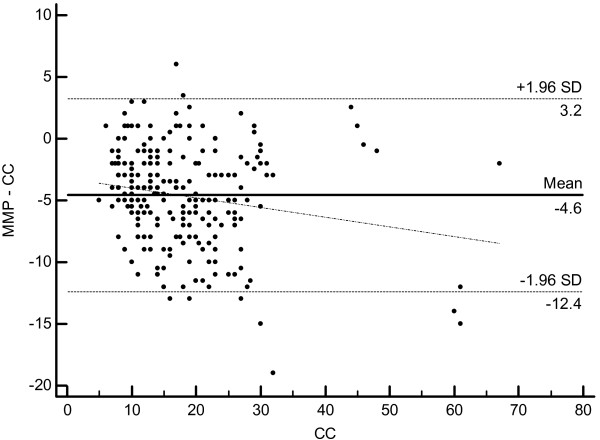
Bland-Altman Plot and 95% limits of agreement of the difference in metatarsophalangeal HV angle from the results of the margo medialis pedis method and the centre head (CC) method.

**Figure 8:**
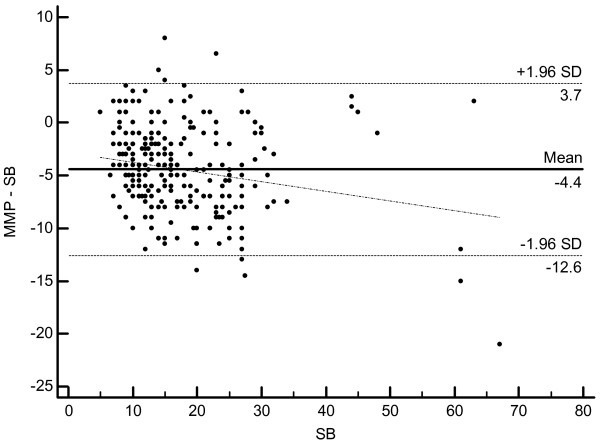
Bland-Altman Plot and 95% limits of agreement of the difference in metatarsophalangeal HV angle from the results of the margo medialis pedis method and the shaft bisection (SB) method.

All three methods had a very good performance, with reliability concordance correlation coefficient (CCC > 0.95). The margo medialis pedis method had the highest reliability and the lowest coefficient of variation. However, the margo medialis pedis method consistently underestimated the hallux valgus angle both, when compared to the centre base – centre head (CC) method (4.8°) or the shaft bisection (SB) method (4.6°) (Table 1). This is also shown in Figures 5 and 6 summarizing the results of Deming regression. Estimating hallux valgus angles based on measurements of the metatarsophalangeal angle (CC and SB methods) by the margo medialis pedis method is associated with low random bias (Table 2). For both methods the CCC coefficient is 0.79 (Cl 0.75 - 0.83). However, despite high precision the accuracy is lower and totally due to systematic bias of 4.8° that was constant over the whole range of measurements. 95% limits of agreement (LA) between the MMP and CC hallux angle measures were -12.6 and 3.2 and between MMP and SB hallux angle measures -12.6 and 3.7. Bland and Altman Plots are given in the Figures 7 and 8. The relatively high negative LA values are due to the systematic hallux valgus underestimation (~4.8°) by the margo medialis pedis method as described above.

**Table 2:** Prediction of the metatarsophalangeal HV angle from the results of the margo medialis pedis method

**Prediction of**	**CCC**	**95% CI**	**Precision**	**Accuracy**	**Shift**	**95% CI**
**CC**	0.79	0.75 – 0.83	0.90	0.88	4.8	2.9 – 6.7
**SB**	0.79	0.75 – 0.83	0.89	0.89	4.8	2.8 – 6.8

Concordance correlation coefficient (CCC) and 95% confidence interval (CI), precision, accuracy and shift (absolute bias) with its 95% CI from estimating results of the centre base – centre head (CC) method and the shaft bisection (SB) method from results of the margo medialis pedis method.

**Figure 9:**
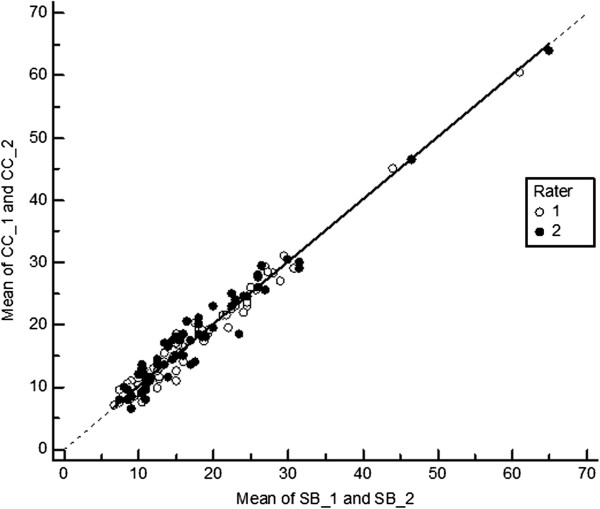
**Deming regression of hallux valgus angles measured by the centre base – centre head (CC) method on measurements based on the shaft bisection (SB) method – measurements 1 and 2 – for both observers (solid line).** The dotted line indicates regression line for unbiased measurements.

The two metatarsophalangeal angle measurement methods (CC and SB) are unbiased estimates of each other. This may be clearly seen in the results of the Deming regression analysis (Figure 9). There is a slightly lower coefficient of variation for the CC method indicating lower random error. This is due to a somewhat higher inter- and intraobserver reliability of the centre base – centre head method.

Table 3 summarizes the intra- and interrater reliabilities for the three measurement methods. All concordance correlation coefficients were above CCC 0.96 and as indicated by the confidence intervals significant at the 0.001 level. (Table 3) The highest overall and intra- as well as interobserver reliability were observed for the MMP method (CCC intrarater 0.988, CCC interrater 0.992).

**Table 3:** Intra- and interrater reliability for the three measurement methods

**Method**	**Intrarater CCC**	**95% CI**	**Interrater CCC**	**95% CI**
**MMP**	0.988	0.983 – 0.991	0.992	0.988 – 0.994
**CC**	0.973	0.962 – 0.981	0.971	0.959 – 0.979
**SB**	0.963	0.948 – 0.974	0.962	0.946 – 0.973

Intra- and interrater concordance correlation coefficients (CCC) and 95% confidence intervals (CI).

## Discussion

The objective of this study was to evaluate the radiographic assessment of the hallux valgus angle of the margo medialis pedis (medial border of the foot) as an alternative to the metatarsophalangeal hallux valgus angle. Basically the hallux valgus angle of the margo medialis pedis can be derived from any method which provides a clear picture of the foot outline without any distortion. Besides non radiographic methods like foot prints, foot scans or foot outline drawings, which are potential methods for screening purposes or preventive interventions, the hallux valgus angle of the margo medialis pedis can also be measured on x-ray images given that the soft tissue of the foot is sufficiently represented. In this study the hallux valgus angle from the margo medialis pedis and the metatarsophalangeal hallux angle were taken from the same x-ray pictures. This gives the opportunity to measure the metatarsophalangeal angle and the hallux valgus angle of the margo medialis pedis, under identical conditions (weight-bearing, foot position, temperature).

The three hallux valgus measuring methods used in the present study have a very good performance with reliability coefficients above 0.95 (p = 0.001) and low coefficients of variation (Table 1). For all methods intra- and interrater concordance correlation coefficients are higher than 0.96 (p = 0.001). Estimating the results of the metatarsophalangeal methods (SB and CC) from the results of the margo medialis pedis method (MMP) is associated with low random bias, high precision (>0.89) and high accuracy (>0.88) (Table 2). Results are nearly identical for SB and CC method. However, there was a systematic bias of 4.8 degrees, constant over the whole range of measurement for both methods. The hallux valgus angle of the margo medialis pedis is consistently smaller than the metatarsophalangeal angle.

We assume that this systematic bias is due to the anatomical varus position of the metatarsale 1 in respect to the os cuneiforme and os naviculare, which has direct impact on the metatarsophalangeal angle but does not affect the hallux valgus angle of the margo medialis pedis. Further studies will be necessary to prove this assumption.

Contrary to a study by Resch [9], in which an interobserver measurement difference of 6.4 degrees was observed in the measurement of the metatarsophalangeal angle, results of the current study show that independent observers arrived at the same results. The interobserver reliability coefficients were higher than 0.96 for all three measurement methods. There was no essential difference between the methods in this respect, though the reliability was a little higher in the margo medialis pedis method. We assume that the high reliability in our study is due to the great emphasis placed on exact measurement definition, the standardised analysis guidelines and the comprehensive pre-experience of the observers with these measurement methods.

A number of different methods for measuring the metatarsophalangeal angle have been published to date [5-10]. The relevant literature gives standard values for the metatarsophalangeal angle, and for the first and second intermetatarsal angles [12,22-24].

The influence of the method on the angle values measured was first discussed by Barnicot and Hardy [13], and confirmed by Schneider and Knahr [12]. For this reason we used two methods for the evaluation of the metatarsophalangeal angle: The shaft bisection method [10], a standard method, and a slight variation of the centre base - centre head method [6] with a detailed advice for the construction of the reference point. The reference point construction disregards the cartilaginous joint surface area on the metatarsal head and therefore can also be identified on postoperative x-ray images after removal of the medial exostosis.

Both methods show high reliability and low variability. The variability of the CC method is lower than that of the SB method and the reliability higher probably due to the more strict definition of the measurement procedure for CC method. Data of this study indicate that the two methods (SB and CC) are unbiased estimates of each other (Table 3, Figure 9). Further studies would be necessary to prove how far the expected advantage of the CC method for pre/post operative hallux valgus angle comparisons holds true.

A limitation of the study is that only patients with a symptomatic hallux valgus deformity were included. It should be mentioned that a direct transfer of our results to non-radiographic material necessary for serial screening investigations is not yet possible.

The main aim of this study was to get basic information about the possibility to use the hallux valgus angle from the margo medialis pedis as an alternative to the metatarsophalangeal hallux angle. We prefered to use the same x-ray pictures for both measurement methods as only this guaranties identical external influences on the hallux position. Though systematic differences between margo medialis measurements from radiographic and non-radiographic pictures are unlikely further investigations necessary. In a preliminary additional study we found very similar hallux valgus values from margo medialis pedis measurements based on foot outline drawings and x-ray pictures. 14 feet from staff members were investigated by two raters two times each. Hallux valgus measures varied from 4° to 37,4° for foot outline drawings and from 0° to 37° for x-ray pictures. T-test showed no significant differences between HV from foot outline drawings and x-ray pictures. (t = 1.7, df = 13, p < 0.5) Correlation between methods as well as intra - and interrater reliability were significant at the p = 0.001 level. (methods r = 0.880; intrarater r = 0.991; interrater r = 0.997) It may be assumed that further results will show that the margo medialis pedis hallux valgus angle derived from non-radiological material measurements is suitable for epidemiological screening of the hallux valgus angle.

## Conclusions

The results of the present study confirm our assumption of a strong-moderate correlation between the hallux valgus angle measured on the margo medialis pedis and the metatarsophalangeal angle when measured from the same x-ray picture. The method shows good performance with highly significant intra- and interrater reliability. Due to a constant bias of -4.8 degrees over the whole measurement range the hallux valgus angles derived from margo medialis pedis method should be considered as conservative estimates of metatarsophalangeal hallux valgus angle. It is to be expected that similar results will be obtained when non-radiographic material (foot outline drawings, foot prints, foot scans) is used for the assessment of the margo medialis pedis hallux angle. But further investigations are necessary.

## Competing interests

The authors declare that they have no competing interests. The study received no financial support.

## Authors’ contributions

EGK was responsible for the overall design of the study. CK was responsible for the acquisition of the radiographic images. Definition of measurement procedures and data acquisition: EGK, WK, CK, AZ; Analysis and interpretation of Data: EGK, MK; All authors helped to draft the manuscript, read and approved the final manuscript.

## Pre-publication history

The pre-publication history for this paper can be accessed here:

http://www.biomedcentral.com/1471-2474/15/133/prepub
